# Targeting extracellular matrix stiffness and mechanotransducers to improve cancer therapy

**DOI:** 10.1186/s13045-022-01252-0

**Published:** 2022-03-24

**Authors:** Yangfu Jiang, Hongying Zhang, Jiao Wang, Yongliang Liu, Ting Luo, Hui Hua

**Affiliations:** 1 Laboratory of Oncogene, State Key Laboratory of Biotherapy, National Clinical Research Center for Geriatrics, West China Hospital, Sichuan University, Chengdu, China; 2 School of Basic Medicine, Chengdu University of Traditional Chinese Medicine, Chengdu, China; 3 Cancer Center, West China Hospital, Sichuan University, Chengdu, China; 4 Laboratory of Stem Cell Biology, West China Hospital, Sichuan University, Chengdu, China

**Keywords:** Cancer, Cancer therapy, Drug resistance, Extracellular matrix, ECM stiffness, Mechanotransducer, Piezo

## Abstract

Cancer microenvironment is critical for tumorigenesis and cancer progression. The extracellular matrix (ECM) interacts with tumor and stromal cells to promote cancer cells proliferation, migration, invasion, angiogenesis and immune evasion. Both ECM itself and ECM stiffening-induced mechanical stimuli may activate cell membrane receptors and mechanosensors such as integrin, Piezo1 and TRPV4, thereby modulating the malignant phenotype of tumor and stromal cells. A better understanding of how ECM stiffness regulates tumor progression will contribute to the development of new therapeutics. The rapidly expanding evidence in this research area suggests that the regulators and effectors of ECM stiffness represent potential therapeutic targets for cancer. This review summarizes recent work on the regulation of ECM stiffness in cancer, the effects of ECM stiffness on tumor progression, cancer immunity and drug resistance. We also discuss the potential targets that may be druggable to intervene ECM stiffness and tumor progression. Based on these advances, future efforts can be made to develop more effective and safe drugs to interrupt ECM stiffness-induced oncogenic signaling, cancer progression and drug resistance.

## Introduction

The extracellular matrix (ECM) is a general scaffold to maintain tissues and organs homeostasis [1]. It is also a critical component of cancer microenvironment that supports tumorigenesis [2]. During tumor development and progression, the complex ECM network is established by fibrillar or non-fibrillar collagens, elastin, proteoglycans, glycoproteins, laminins, fibronectins and other matrix proteins. ECM not only provides nests for cancer and stroma cells, but also serves as a reservoir for growth factors and cytokines. Furthermore, ECM interacts with neighboring cells and initiates diverse cellular signaling cascades to promote tumor growth and metastasis. Collagens are the main components of ECM. Previous studies have demonstrated that many collagen proteins are overexpressed in human tumors, and correlated with poor prognosis in cancer patients [3]. While there are many collagen genes, *ELN* is the only gene encoding the elastin precursor tropoelastin in humans [4]. Extracellular tropoelastin aligns on microfibrils scaffold and then assembles into elastic fibers [4]. Except for collagens and elastin, the high molecular weight polymer hyaluronan and its fragments play important roles in cancer development and progression by remodeling the tumor microenvironment and reprogramming cancer metabolism [5, 6]. Other substances within the ECM, such as laminins and fibronectins, also are critical for tumorigenesis [7, 8]. The diverse components in the tumor ECM work in concert to promote tumor growth, invasion and metastasis. These ECM components can be potential prognostic biomarkers and therapeutic targets for cancer.

The ECM is a complex structure that is dynamically remodeled by the synthesis and degradation of ECM proteins [9]. Cleavage of ECM components by matrix metalloproteinases (MMPs), adamalysins and meprins is an important mechanism of dynamic regulation of ECM abundance and structure [10]. During tumorigenesis, ECM breakdown may lead to the release of growth factors and cytokines that are sequestered by ECM, thereby inducing tumor cells growth, angiogenesis and inflammation. On the other hand, a change in the abundance of ECM components contributes to different tissue density and stiffness. Accumulating evidences demonstrate that mammographic density is positively associated with breast cancer risk [11]. Matrix stiffening also contributes to increased cancer risk in fibrotic organs [12]. Indeed, ECM stiffening alone can induce the malignant transformation of mammary epithelial cells [13]. Similar effects of matrix stiffness are also detected in the carcinogenesis of liver, pancreas and other tissues [14–16]. Increased ECM stiffness may be another hallmark of cancer. While the ECM stiffness in the brain, lung, breast or pancreas is usually less than 1000 Pa, it may reach 4–10 kPa in tumors at these sites [17].

Matrix stiffness is tightly regulated by cancer microenvironment such as hypoxia [18, 19]. Meanwhile, systemic health problem such as obesity may affect breast adipose microenvironment and raise the matrix stiffness [20]. Matrix stiffening generates mechanical cues that act on stromal cells, parenchymal cells, premalignant cells or cancer cells, and stimulate cell transdifferentiation, autophagy, epithelial-mesenchymal transition (EMT), cell migration, invasion and metabolic reprogramming [21, 22]. Given that matrix stiffening may increase the risk of cancer development and progression, pharmacological intervention in matrix stiffness is emerging as an option for cancer prevention and treatment. A better understanding of the mechanisms underpinning the regulation of tumorigenesis by matrix stiffness is critical for identifying druggable targets in this process. Herein, we introduce recent advances in identifying the regulators of matrix stiffness and summarize the progresses in elucidating the mechanisms underpinning the promotion of tumor development, progression and drug resistance by matrix stiffening. Based on these conceptual advances, we discuss what ECM stiffness-related targets may be of therapeutic potential for cancer patients. Insight into the matrix biology may inspire better therapeutic approaches for cancer.

## The regulators of ECM stiffness

**Fig. 1:**
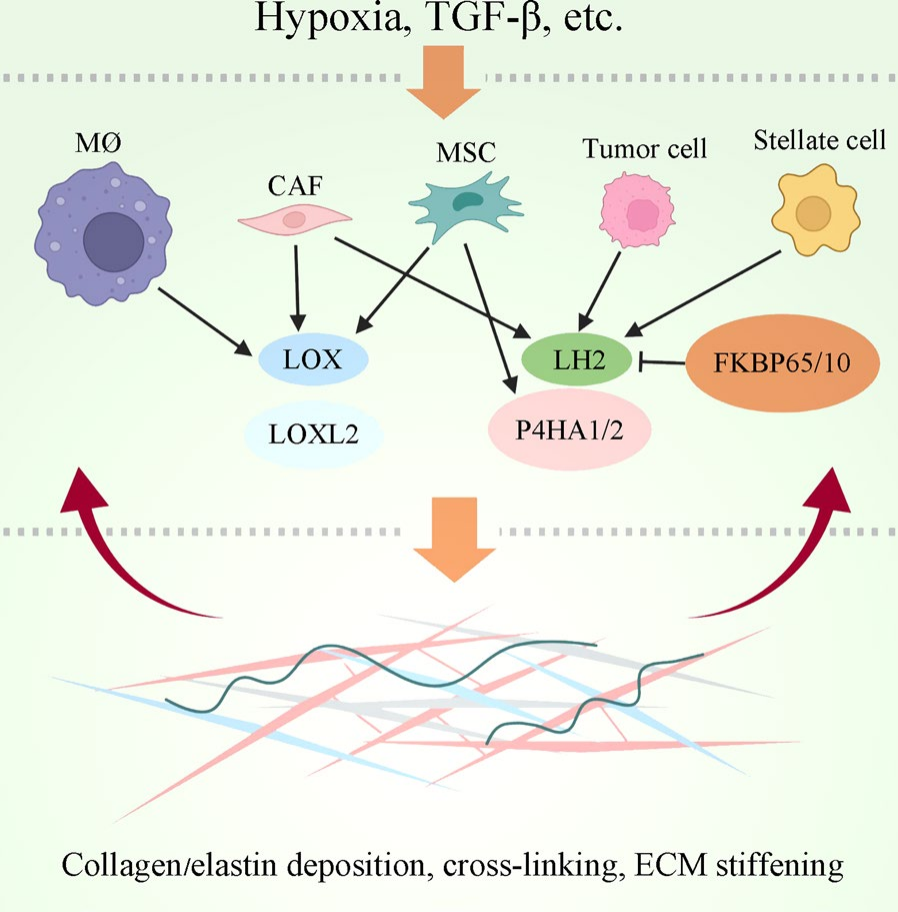
The regulation of ECM stiffness by tumor and stromal cells. Hypoxia or growth factors such as TGFβ can induce the expression of collagen/elastin cross-linking factors in tumor and stromal cells, leading to increased ECM stiffness. ECM stiffening reciprocally acts on tumor and stromal cells thereby generating a vicious cycling. M∅, macrophage; CAF, cancer-associated fibroblast; MSC, mesenchymal stem cell

The ECM proteins collagens and elastin are critical contributors to ECM stiffness. Activation of many key signaling pathways such as TGFβ, insulin-like growth factor (IGF)/IGF1R and PI3K/Akt can promote the synthesis of ECM proteins [23–27] (Fig. 1). The endoplasmic reticulum-resident protein Hsp47 is a molecular chaperone that promotes procollagens folding and processing. Increased Hsp47 expression may enhance the secretion of collagens into the ECM, thereby promoting collagens deposition. In addition, Hsp47 interacts with decorin, lumican and fibromodulin to promote their secretion into the ECM [28]. Secreted proteome acidic and rich in cysteine (SPARC) is another matrix chaperone that binds collagens in the ECM, prevents collagens degradation, and facilitates correct collagens assembly [29]. Hence, both intracellular and extracellular chaperones are involved in the regulation of ECM proteins secretion and deposition.

Site-specific post-translational modification of collagen is critical for the solubility and alignment of collagen. Both the density and the alignment of collagens or elastin are critical determinants of ECM stiffness. Collagens/elastin cross-linking and the highly organized matrix fibers are responsible for matrix stiffening [30, 31]. Stromal cells-secreted lysyl oxidases (LOX) are the major enzymes that catalyze covalent cross-linking of both collagens and elastin [32–34]. Mechanistically, LOX-catalyzed oxidative deamination of lysine and hydroxylysine residues in collagen and elastin precursors generates allysine residues that react with other allysine or lysine residues to form cross-links [4]. The fibrogenic messenger TGF-β1 can induce LOX expression in diseases such as cancer. In addition, lysyl hydroxylase 2 (LH2) specifically hydroxylates lysine residues in collagen telopeptides, which is critical for the formation of stabilized cross-links [35]. Either tumor cells- or cancer-associated fibroblasts (CAFs)-secreted LH2 induces hydroxylysine aldehyde-derived collagen cross-links in tumor stroma and then increases tumor stiffness [22, 36, 37]. LH2 is frequently overexpressed in various types of cancer. The transcription factors HIF1A, SMADs and GATA3 directly induce LH2 expression [18, 35, 38]. Moreover, FK506 binding protein (FKBP) 65, a peptidyl-prolyl cis–trans isomerase, interacts with LH2 and promotes its dimmerization, thereby enhancing collagen pyridinoline cross-linking [39]. FKBP10 also promotes collagen cross-linking by interacting with LH2 [40]. Besides, collagen cross-linking is regulated by tissue transglutaminases [41]. The joint promotion of collagen cross-linking by LOX and transglutaminase synergistically increases tissue stiffness.

Furthermore, overexpression of collagen prolyl 4-hydroxylase alpha-1/2 (P4HA1/2) in cancer cells and fibroblasts may increase collagen deposition [18, 42]. LH2 co-operates with P4HA1/2 to increase matrix stiffness by enhancing the alignment of deposited collagen fiber [18]. In addition, the stellate cells in some tissues, such as pancreatic and hepatic stellate cells, contribute to hypoxia-induced matrix stiffening by overexpression of LH2 [31]. HIF1A act as a master regulator of LOX, P4HA1/2 and LH2 to mediate the regulation of matrix stiffness by hypoxia [43]. On the other hand, matrix stiffening can promote hepatic stellate cells differentiation into myofibroblasts that produce matrix proteins, resulting in a vicious cycling [44]. Activated hepatic stellate cells also produce periostin, which is capable of up-regulating LOX and LOXL to facilitate matrix stiffening [45]. In contrast, fibronectin negatively regulates liver fibrosis and matrix stiffness by inhibiting hepatic stellate cells activation and response to TGFβ [46]. Hence, different matrix proteins may positively or negatively regulate ECM stiffness.

Rho-GTPases are members of the Ras homology proteins family. Rho-associated protein kinase (ROCK) is another mediator of the cross-talk between tumor cells and microenvironment [47]. While Rho kinase (ROCK) is a mechanosensor of matrix stiffness, it also feed-forwards to increase tissue stiffness through β-catenin-mediated synthesis of collagen, fibronectin and periostin [48, 49]. In addition, ROCK2 inhibits p21 expression but enhances NF-kB and tenascin C expression, indicating the up-regulation of tissue rigidity by ROCK2 [50]. However, one study indicates that treatment of Kras^G12D^/p53^R172H^ mice with a ROCK inhibitor leads to increased collagen in pancreatic ductal adenocarcinoma [51]. It remains unclear how to interpret these inconsistent roles of ROCK in regulating ECM stiffness. Further studies are warranted to address this issue.

In addition, matrix stiffness is regulated by oncogenes and tumor suppressor genes. The transcription factors Twist1 and ZEB1 are powerful oncogenes that promote EMT and cancer metastasis. ZEB1 can up-regulate LOX and LOXL2 expression by inhibiting miR-200, thereby promoting collagen cross-linking and matrix stiffening [52]. While the roles of Twist1 in cancer cells are well studied, little is known about the involvement of Twist1 in tumor stroma cells. Overexpression of Twist1 not only promotes the fibroblasts-CAFs transition, but also increases matrix stiffness by promoting the expression of collagen type VI α1 chain in CAFs [53]. In addition, Twist1 may act as a mechanoresponser to matrix stiffness [54]. High matrix stiffness leads to the release of Twist1 from GAP SH3 domain-binding protein 2 (G3BP2), thereby promoting Twist1 nuclear translocation and enhancing EMT in tumor cells [54]. These studies collectively demonstrate that Twist1 integrates the matrix stiffness-mediated cross-talk between CAFs and tumor cells.

In the tumor microenvironment, senescent mesenchymal stem cells (MSCs) may increase collagen density and matrix stiffness [55]. On the other hand, tumor stiffness reciprocally regulates MSCs differentiation and reprograms mesenchymal stromal cells to enhance their pro-tumorigenic activities [56]. Besides, many growth factors can stimulate ECM stiffness. Except for TGFβ, activation of platelet derived growth factor receptor-alpha in mammary fibroblasts leads to increased hyaluronic acid and collagen deposition in the mammary fat pad, thereby increasing mammary stiffness [57]. The interplay between ECM stiffness and growth factor signaling is critically involved in cancer progression, immune surveillance and drug resistance.

## The regulation of tumor growth and metastasis by matrix stiffness

**Fig. 2:**
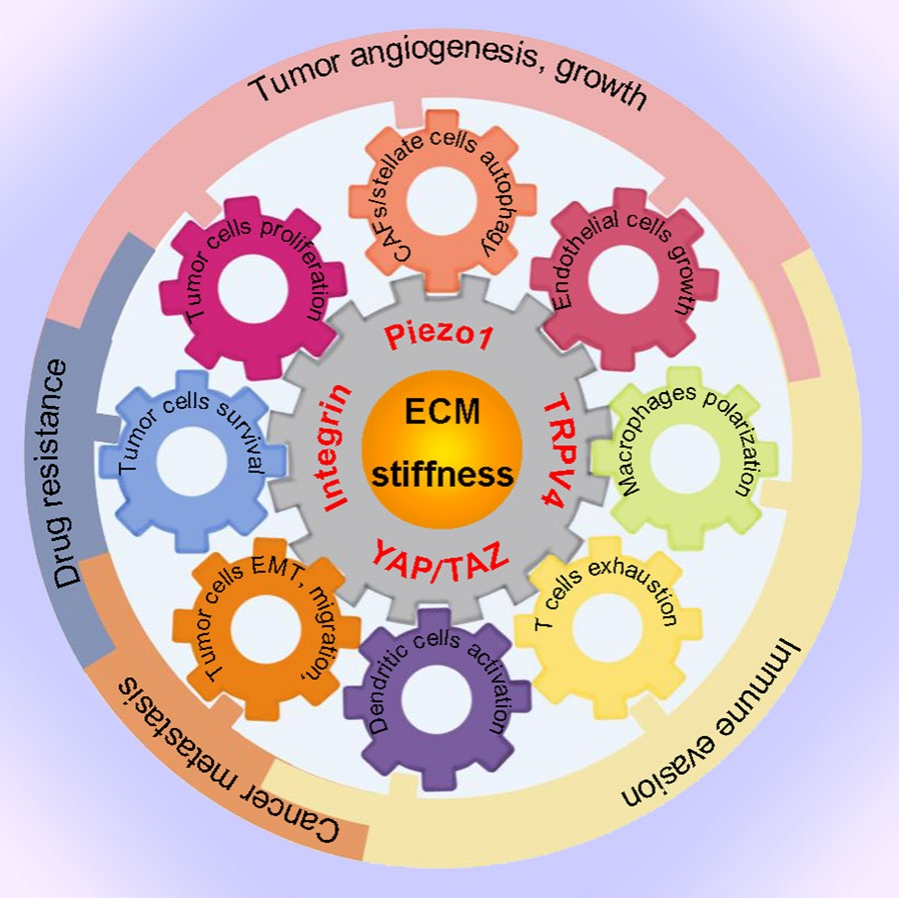
Regulation of tumor angiogenesis, growth, metastasis, immune evasion and drug resistance by ECM stiffness. ECM stiffening-induced mechanical cues drive tumor cells proliferation, CAFs/stellate cells autophagy and endothelial cells growth, thereby stimulating angiogenesis and tumor growth. ECM stiffening also promotes cancer metastasis by inducing EMT and cancer cells migration. The promotion of macrophage polarization and T cells exhaustion by ECM stiffening contributes to immune evasion in cancer

Matrix stiffness may change the mechanical properties of tissues and tumors. Tumor cells and stromal cells can respond to the matrix stiffening-induced mechanical signal by mechanosensors or mechanotransducers. ECM stiffening typically induces mechanical perturbations of the lipid bilayer and activation of the TRP (transient receptor potential) family channels and Piezo channels, the evolutionarily conserved ion channels linking ECM stiffening-related mechanical force to cell signaling pathways, especially the Ca^2+^-signaling in tumor and stromal cells [58–60]. The transmembrane receptor integrin, which can promote cancer stemness and drug resistance, is a mechano-signal transducer that can be activated by Piezo [61, 62]. The physical interaction between the extracellular domain of integrins and ECM proteins induces the assembly of cytoplasmic complexes consisting of scaffold proteins (vinculin, talin, paxillin, etc.), focal adhesion kinase (FAK), Src and PI3K/Akt, thereby coordinating focal adhesion and cytoskeleton assembly with matrix mechanical cues [63]. Rap1 GTPase also responds to matrix stiffening by stabilizing integrins and recruiting vinculin to focal adhesions [64]. In addition, ROCK activation may be induced by ECM stiffening and then promotes integrin signaling, MAPK activation and SNAIL stabilization [65–67]. Integrin, integrin-linked kinase (ILK), SNAIL and Src also up-regulate the expression and activation of YAP, another critical mechanotransducer that can feed-forward to up-regulate Piezo1 expression [66, 68–71]. Of note, YAP does not always respond to stiff ECM [72]. The ECM stiffening-induced diverse signaling in tumor and stromal cells may promote tumor growth, angiogenesis, metastasis, immune evasion and drug resistance (Fig. 2).

### Stimulation of tumor growth by ECM stiffening

As described above, mammographic density is critically correlated with the development of breast cancer. Previous studies have uncovered many mechanisms underlying the promotion of mammary tumorigenesis by increased mammary stiffness. Matrix stiffness switches prolactin signals from physiological STAT5 activation to protumorigenic Src/FAK and MMP activation and promotes the protumorigenic cross-talk between estrogen and prolactin in breast cancer cells [73, 74]. In addition, FAK-Rho-ERK signaling is involved in the promotion of mammary epithelial cells growth by matrix stiffness-induced mechanical stimuli [75]. ECM stiffness also stimulates mammary epithelial cells proliferation by down-regulating miR-203 expression and up-regulating ZNF217-mediated Akt activation [76]. On the other hand, ECM stiffness may indirectly promote breast cancer cells proliferation by enhancing mesenchymal stem cells differentiation into CAFs [77].

Stellate cells are associated with fibrosis in liver and pancreas. Matrix stiffness may induce fibroblasts or stellate cells autophagy through integrin- and FAK-mediated stabilization of AMPKα at focal adhesions, which promotes adjacent cancer cells growth [78]. Meanwhile, activation of RhoA-Akt-P300 axis by ECM stiffness promotes the differentiation of hepatic stellate cells into myofibroblasts that enhance the outgrowth of metastatic liver cancer [14]. Angiogenesis is important for sustained tumor growth. ECM stiffness stimulates tumor angiogenesis by promoting the activation of splicing factors and then increasing the production of protein kinase C (PKC) βII and the extra domain-B splice variant of fibronectin in endothelial cells [79, 80]. Furthermore, stiff ECM may promote nucleotide synthesis and tumor growth by preventing LATS1/2- and TRAF2-mediated degradation of phosphoribosyl pyrophosphate synthetase 1/2 [81]. Together, these studies demonstrate that ECM stiffness may promote tumor growth by jointly regulate both tumor and stromal cells.

### Stimulation of cell migration and cancer metastasis by ECM stiffening

While ECM is supposed to be a barrier for cell migration, cancer cells or cancer-associated fibroblasts may secret proteases to remodel the ECM and break through the barrier. On the other hand, ECM can provide migration tracks to facilitate directional cancer cell migration [82]. As described above, ECM stiffening-induced mechanical stimuli may lead to increased actomyosin contractility in neighboring cells. Upon ECM stiffening, increased actomyosin contractility results in the activation of RhoA-mDia1 signaling and microtubule network remodeling, which allows adenomatous polyposis coli protein to recruit a set of RNAs to the contractile protrusions and promotes cell migration [83].

On the other hand, ECM stiffening can activate the mechanosensor Piezo1, which is a mediator of mechanical force-induced cancer metastasis [84]. Downstream of integrin and Piezo1, YAP activation may promote cell migration by stimulating aerobic glycolysis and MMP-7 expression [85, 86]. Besides, transient receptor potential vanilloid 4 (TRPV4) is another mechanosensitive ion channel that may act as a sensor of ECM stiffness [87]. TRPV4 can promote matrix stiffness-induced EMT by enhancing Akt activation and YAP/TAZ translocation into the nucleus [88].

ECM stiffness-induced mechanical forces also down-regulate ubiquitin domain-containing protein 1 expression or redistribute ubiquitin domain-containing protein 1 to cell–cell contacts and prevent the association between the E3 ubiquitin ligase β-TrCP and YAP1, thereby suppressing YAP1 degradation and facilitating ROCK2-dependent YAP1 activation, EMT, cancer cells migration and invasion [89, 90]. In addition, the stimulation of EMT by ECM stiffness may be mediated by Twist1 and discoidin domain receptor 2, which is up-regulated by p300-c-Myb-LEF1 axis [54, 91].

Ephrin receptor is another cell membrane protein that mediates ECM stiffness-induced EMT and cancer metastasis. Ligand-independent activation of ephrin receptor EPHA2 by matrix stiffening leads to LYN kinase-mediated Twist1 phosphorylation and nuclear translocation, thereby promoting EMT, cancer cells invasion and metastasis [92]. Matrix stiffness promotes liver cancer metastasis by integrin- and TGFβ-mediated up-regulation of Snail [21]. Furthermore, the metastatic potential of cancer cells may be heterogenous in a microenvironment with stiff ECM. Cancer cells with increased viscosity have greater invasive potential [93].

While a stiff ECM may promote cancer progression by integrin-, FAK- and YAP/TAZ-mediated signaling, it is also reported that a soft ECM can stimulate cell invasion by inhibiting cell adherence and upregulating the secretion and activation of MMP [94]. Moreover, depletion of the epithelial cell-associated vacuolar ATPase ‘a2’ isoform in mammary gland renders breast tumors being soft but highly metastatic [95]. Although the defective ECM glycosylation and cross-linking may be responsible for the low ECM stiffness in this model, it is still unclear whether other ECM stiffness-independent effects contribute to the pro-metastasis effect of epithelial cell-associated vacuolar ATPase ‘a2’ isoform depletion. Nevertheless, it warrants further studies to uncover how cancer cells may adapt to changes in the ECM stiffness.

## The promotion of cancer drug resistance by ECM stiffening

Since the EMT program is a critical contributor to anticancer drug resistance [96], it is not surprising that ECM stiffness may regulate the response to cancer therapy. Previous study has demonstrated that ECM stiffness induces EMT and paclitaxel resistance in pancreatic cancer [97]. Of note, many mechanisms may be involved in the regulation of cancer drug resistance by ECM stiffness. The long noncoding RNA nuclear paraspeckle assembly transcript 1 is responsive to a stiff ECM, leading to increased paraspeckle that contributes to chemotherapy resistance [72, 98]. In addition, the triple negative breast carcinoma cells MDA-MB-231 exhibit ECM stiffness-dependent resistance to doxorubicin due to YAP activation [99]. Also, the sensitivity of hepatocellular carcinoma and ovarian cancer cells to platinum therapy can be reduced by a stiff ECM through integrin-, FAK-, Akt-, STAT3- and YAP-dependent mechanisms [100, 101]. Another mechanism of ECM stiffness-dependent sensitivity to genotoxic drugs involves DNA double-strand breaks repair efficiency [98]. The activity of MAP4K4/6/7 is higher in soft ECM-surrounded cancer cells compared with stiff ECM-neighboring cells, which results in elevated ubiquitin phosphorylation, impaired ubiquitin signaling at DNA double-strand breaks sites, DNA repair deficiency and increased sensitivity to genotoxic agents [102]. However, another study indicates that a stiff ECM may sensitize triple negative breast carcinoma cells to chemotherapy by enhancing proapoptotic JNK activity, while triple negative breast carcinoma cells surrounded by a soft ECM may be resistant to chemotherapy as a result of elevated NF-κB activity and decreased JNK activity [103]. This paradigm highlights the plasticity of cancer cells in adaptation to changes in ECM stiffness.

Metformin is an anti-diabetes drug that also has anticancer effects [104]. ECM stiffening compromises the up-regulation of PTEN and down-regulation of Akt activity by metformin, leading to metformin resistance [105]. Moreover, ECM stiffness can affect the sensitivity of cancer cells to molecular-targeted agents. Sorafenib is one of the first-line systemic therapies for advanced hepatocellular carcinoma [106]. The sensitivity of hepatocellular carcinoma cells to sorafenib is reduced in a stiff microenvironment, due to the activation of integrin-JNK signaling [107]. As described above, tissue stiffness can promote angiogenesis [79]. Recent study also demonstrates that increased ECM stiffness in colorectal liver metastasis may enhance anti-angiogenic therapy resistance [108]. Besides, increased ECM stiffness reportedly promotes breast cancer cells resistance to the HER2 inhibitor lapatinib and melanoma cells resistance to the BRAF inhibitor vemurafenib [109, 110]. Collectively, these studies demonstrate that ECM stiffness is a determinant of the response to pathways-targeted anticancer agents.

## The regulation of cancer immunity by ECM stiffness

Immune checkpoints blockade is another treatment for cancer [111, 112]. Evasion of the immune surveillance is critical for tumor growth. The mechano-signal transducers Piezo1, integrin and YAP are tightly involved in cancer immunity [113, 114]. Given that the mechanotransducer integrin may activate TGFβ and promote immune evasion [114], ECM stiffening may promote immune evasion in cancer. Programmed death-ligand 1 (PD-L1) can help cancer cells resist immune surveillance. Stiff ECM may enhance PD-L1 expression in cancer cells. ECM stiffening-induced increase in extracellular pressure often leads to hypoxia. It is unclear whether the up-regulation of PD-L1 by stiff matrix is dependent on YAP/TAZ or HIF1A, which positively regulates PD-L1 expression [115–117]. Together with autocrine TGFβ signaling, high collagen density and ECM stiffening may reduce the abundance and function of cytotoxic T cells in tumors [118]. Besides, high collagen density and Piezo1 activation may promote macrophages polarization and enhance their immunosuppressive phenotype, leading to reduced cytotoxic T cells abundance and proliferation [113, 119, 120]. ECM stiffness is negatively correlated with T cells infiltration in tumors and the efficacy of PD1 blockade therapy [121]. Meanwhile, collagen may directly promote CD8^+^ T cells exhaustion through interacting with CD17 and LAIR1 [122]. Hence, high collagen density may directly or indirectly promote immune evasion and immunotherapy resistance in cancer. The regulation of other immune checkpoints by ECM stiffening remains to be studied.

While the above-mentioned studies suggest that Piezo1 and integrin may contribute to immune evasion in cancer, other studies also indicate that integrin is critical for T cells activation [123]. In addition, previous studies suggested that Piezo1 might be involved in T cells activation [124]. However, recent study in an animal model of experimental autoimmune encephalomyelitis demonstrates that Piezo1 deletion in T cells does not affect effector T cells function but paradoxically expand the pool of immunosuppressive regulatory T (Treg) cells, suggesting that activation of Piezo1 in T cells may enhance immune response in this autoimmune disease [125]. The same study also finds that deletion of Piezo1 in Treg cells may inhibit immune response [125]. Besides, dendritic cells are critical for activating T cells and evoking an immune response [126]. While increased extracellular pressure may promote dendritic cells maturation and CD4^+^ T cell proliferation [127], one study indicates that increased substrate stiffness appears to hamper the ability of dendritic cells to evoke immune response in vitro [128]. Another study also demonstrates that mechanical stiffening inhibits the migration of dendritic cells [129]. In contrast, one study indicates that an increase in mechanical stiffness may promote the activation of dendritic cells during cancer immunotherapy by activating the mechano-signal transducers Piezo1 and TAZ [130]. Together, these studies indicate that the roles of ECM stiffness in immunity are complex and immune cell type-dependent. The effects of ECM stiffening on immune surveillance may be dependent on the balance among diverse pathways in different cell types. It warrants further studies to address and clarify the effects of ECM stiffness on immune surveillance and cancer immunotherapy in more relevant in vivo models.

## The targets and drugs for intervention in ECM stiffness

**Fig. 3:**
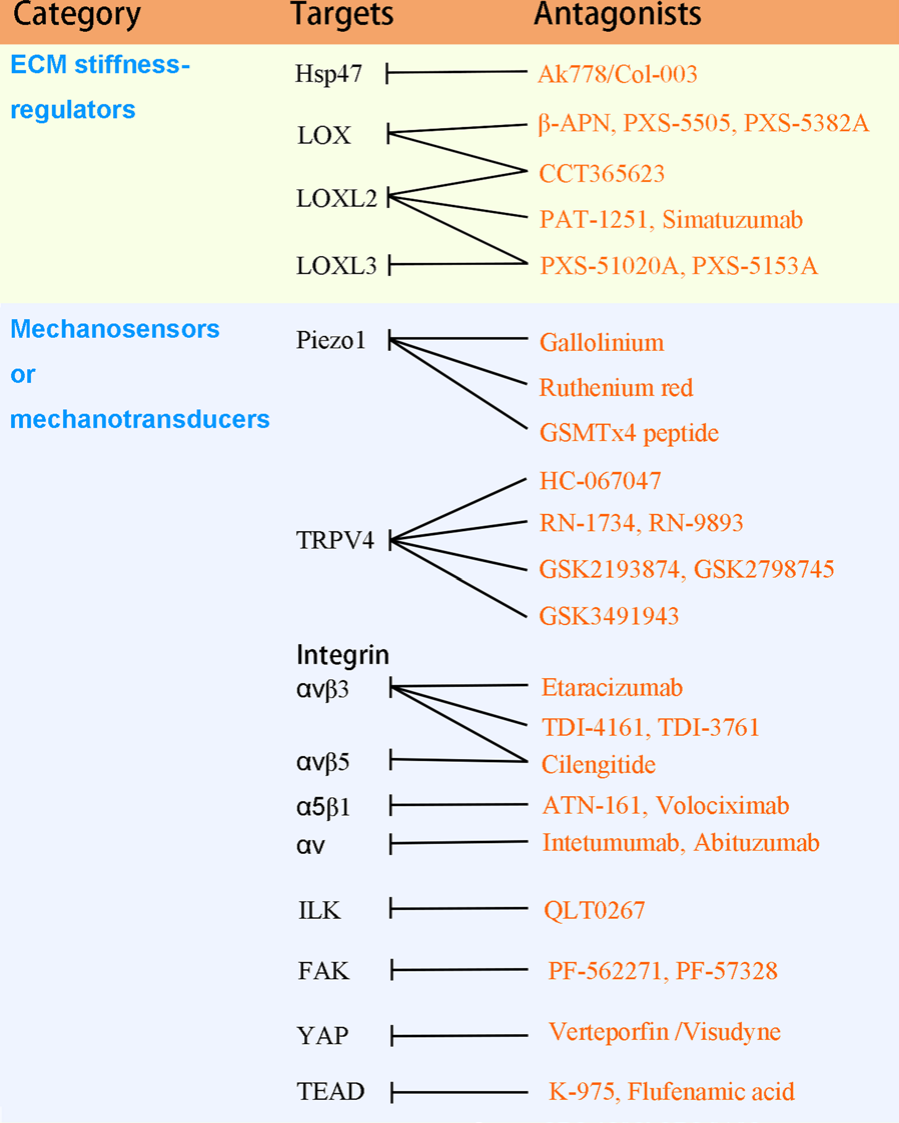
The antagonists of ECM stiffness regulators and mechano-signal transducers. Some of the inhibitors of collagen chaperone Hsp47, lysyl oxidases, mechanosensors Piezo1 and TRPV4, mechanotransducers integrin, ILK, FAK, YAP and TEAD are shown

Since the rigid and cross-linked ECM not only promotes tumorigenesis but also impairs the intratumoral distribution of immune cells and anticancer drugs, targeting ECM stiffness may be a strategy to treat cancer and overcome drug resistance. Previous studies have demonstrated that some of the regulators of ECM stiffness, the mechanosensors and mechanotransducers are druggable. Since fibrillar collagen is a major contributor to increased ECM stiffness, direct depletion of collagen by recombinant collagenase has emerged as potential cancer therapeutics. In addition, the inhibitors of Hsp47, LOX, LOXL2, LOXL3, integrin, Piezo1, TRPV4, ILK, YAP/TAZ and TEAD have been developed (Fig. 3). Many of these inhibitors show anticancer activities in preclinical studies.

### Collagen chaperone-targeted agents

Given the essential roles of Hsp47 in the folding, secretion and assembly of collagens, Hsp47 may be a potential target for the treatment of ECM-related disorders such as fibrosis and cancer. TGFβ can induce the expression of Hsp47. The TGFβ inhibitor pirfenidone can inhibit Hsp47 and collagen expression, which may contribute to the antifibrotic effect of pirfenidone [131]. The small molecule AK778 and its cleavage product Col003 can inhibit the interaction between Hsp47 and collagen, thereby destabilizing the collagen triple helix and suppressing its secretion [132]. Other virtual screening identified some compounds that may interrupt the interaction between Hsp47 and collagen [133]. One of these compounds, methyl 6-chloro-2-oxo-2,3-dihydro-1,2lambda ~ 4 ~ ,3-benzodithiazole-4-carboxylate, shows antifibrotic effect [134]. In addition, preclinical studies have demonstrated that vitamin A-coupled lipid nanoparticles containing siRNA against Hsp47 could inhibit hepatic, pulmonary and pancreatic fibrosis [135, 136]. The safety of ND-L02-s0201 injection, a vitamin A-coupled lipid nanoparticle containing siRNA against Hsp47, has been evaluated in healthy humans and subjects with hepatic fibrosis (Table 1). While the anticancer effect of vitamin A-coupled lipid nanoparticle containing siRNA against Hsp47 is unclear, the PEGylated polyethylenimine-coated gold nanoparticles containing all-trans retinoic acid and siRNA against HSP47 could inhibit ECM deposition, enhance drug delivery to pancreatic tumors and improve chemotherapy efficacy [137]. Overall, the development of Hsp47 inhibitors is still in its infancy. It deserves further studies to determine the safety and efficacy of these identified Hsp47 inhibitors in cancer therapy. While SPARC is also a chaperone for collagens, it has anticancer effects and adipogenesis-inhibiting function [138]. Therefore, it appears that SPARC inhibition is not an appropriate option for cancer therapy.

**Table 1:** The clinical trials of drugs targeting LOX, Hsp47, integrins, YAP1 or TRPV4 (clinicaltrials.gov)

Trial ID	Target	Drug	Combination	Conditions	Phase	Estimated or actual enrollment	Status/results
NCT04676529	Pan-LOX	PXS-5505	None	Myelofibrosis	I/IIa	24	N.A
NCT02852551	LOXL2	PAT-1251	None	Healthy adults	I	78	This compound was well tolerated [140]
NCT04305496	LOXL2	PAT-1251	None	Myelofibrosis	II	21	Recruiting
NCT04183517	LOX	PXS-5382A	None	Healthy adults	I	18	N.A
NCT01369498	LOXL2	Simtuzumab	Ruxolitinib	Myelofibrosis	II	54	Simtuzumab alone or the addition of simtuzumab to ruxolitinib did not have clinical benefit [149]
NCT01472198	LOXL2	Simtuzumab	Gemcitabine	Pancreatic cancer	II	250	The addition of simtuzumab to gemcitabine did not improve clinical outcomes [146]
NCT01858935	Hsp47	ND-L02-s0201 injection	None	Healthy adults	I	56	Completed
NCT00689221	Integrin	Cilengitide	Temozolomide & radiotherapy	Glioblastoma	III	545	The addition of cilengitide to temozolomide chemoradiotherapy did not improve outcomes [161]
NCT04177108	Integrin	ATN-161	Carboplatin	Malignant glioma	I/II	82	Completed
NCT00066196	Integrin	MEDI-522	Dacarbazine	Metastatic melanoma	II	110	Completed
NCT00401570	α5β1Integrin	Volociximab	Gemcitabine	Metastatic pancreatic cancer	II	40	Completed
NCT04590664	YAP1	Verteporfin	None	Glioblastoma	I/II	24	Recruiting
NCT03033225	YAP1	Verteporfin	Photodynamic therapy	Advanced pancreatic carcinoma	II	30	Recruiting
NCT02119260	TRPV4	GSK2798745	None	Healthy subjects and heart failure patients	II	61	This compound is safe and well tolerated [153]

### Preclinical and clinical development of Lysyl oxidase-targeted agents

Given that lysyl oxidases are critical inducers of ECM rigidity, inhibition of lysyl oxidases is a promising approach to reduce matrix stiffness. Both pan-LOX family inhibitors and specific inhibitors of a LOX family member have been developed [139, 140]. These LOX inhibitors exhibit anticancer effects in preclinical studies. β-aminopropionitrile (β-APN) is a pan-LOX inhibitor that can suppress the migration and invasion of breast cancer cells [141]. However, prolonged treatment with β-APN may have adverse effects such as aortic injury and osteolathyrism, which precludes the administration of this compound in clinic. Instead, PXS-5505 is another pan-LOX inhibitor that has been proved safe in phase I clinical trial. A phase I/IIa study has been initiated to evaluate the safety and tolerability of PXS-5505 in patients with primary, post-polycythemia vera or post-essential thrombocythemia myelofibrosis (Table 1). Moreover, some dual LOX/LOXL2 or LOXL2/LOXL3 inhibitors have been developed. The aminomethylenethiophene scaffold-bearing inhibitor CCT365623 is a dual LOX/LOXL2 inhibitor that can suppress breast cancer growth and metastasis [142]. The indole-based fluoroallylamine PXS-51020A and PXS-5153A are LOXL2/LOXL3 inhibitors have antifibrotic activity in preclinical models of liver and lung metastasis [143, 144]. In addition, preclinical studies have demonstrated that PAT-1251/GB2064, a highly selective LOXL2 inhibitor based on a benzylamine with 2-substituted pyridine-4-ylmethanamines, has collagen accumulation-lowering and tumor-suppressing effects [145]. After phase I clinical trial of PAT-1251 demonstrated that it was well tolerated, a phase IIa trial in patients with myelofibrosis has been initiated (Table 1). Another pan-LOX inhibitor PXS-5382A has also been tested in healthy volunteers.

Simtuzumab is a humanized IgG4 monoclonal antibody against LOXL2. Although this drug shows preclinical promise and well tolerability, phase II studies of simtuzumab have demonstrated that it has no clinical benefit in patients with idiopathic pulmonary fibrosis, primary myelofibrosis, post-polycythemia vera myelofibrosis, post-essential thrombocythemia myelofibrosis, advanced fibrosis caused by non-alcoholic steatohepatitis, metastatic pancreatic adenocarcinoma and metastatic *KRAS* mutant colorectal carcinoma [146–149]. So far, the prospect of simtuzumab in cancer therapy remains to be poor.

### Preclinical and clinical development of Piezo1-, TRPV4- or integrin-targeted agents

Except for the regulators of collagen and elastin cross-linking, mechanosensors or mechanotransducers may be alternative targets for intervention of ECM stiffness-induced signaling. The mechanosensor Piezo1 not only mediates the effects of ECM stiffness on cancer cells, but also contributes to ECM rigidity-induced expansion of immunosuppressive myeloid cells [113]. While gadolinium and ruthenium red can inhibit Piezo1, the tarantula venom-derived peptide GsMTx4 is more selective inhibitor of Piezo1 and other cationic mechanosensitive ion channels [150]. Although GsMTx4 can suppress immune evasion in cancer [113], its effects on tumor growth and metastasis remain elusive. It warrants further studies to develop small-molecule inhibitors of Piezo1 or monoclonal antibodies against Piezo1.

Besides Piezo1, another mechano-sensitive ion channel TRPV4 is involved in tumor progression. TRPV4 antagonists, such as HC-067047, RN-1734, RN-9893, GSK2193874, PF-05214030, GSK2798745 and GSK3491943, have been developed in recent years [151, 152] (Fig. 3). Among them, GSK2798745 is the first TRPV4 blocker that has been evaluated in clinical trial. Early phase clinical trial has demonstrated that GSK2798745 is safe and well tolerated in humans [153]. While TRPV4 promotes cancer cells proliferation, migration and extravasation, activation of TRPV4 in endothelial cells suppresses vascular endothelial growth factor signaling, normalizes tumor vasculature, inhibits tumor growth and metastasis, and improves cancer therapy [154, 155]. It is unclear whether the normalization of tumor vasculature is a unique function of TRPV4 but not other mechanosensors. In addition, activation of TRPV4 inhibits glioma by inducing lethal mitophagy [156]. Pharmacological activation of TRPV4 by GSK1016790A also induces melanoma and breast cancer cells death and inhibits breast tumor growth [157, 158]. Hence, the effects of TRPV4 may be cell type- or cancer type-specific. The potential validity of TRPV4 agonists or antagonists in cancer therapy needs more studies. Of note, systemic administration of TRPV4 agonists may have severe effects that preclude clinical application [159].

As mentioned above, integrin is a critical mechano-signal transducer that mediates the tumor-promoting effects of ECM stiffening. Therefore, targeting integrin is an approach to unleash the effects of ECM stiffness on tumorigenesis. Preclinical studies have demonstrated that integrin inhibitors could significantly suppress tumor growth and metastasis [160]. Cilengitide is a selective αvβ3/αvβ5 integrin inhibitor that has been assessed in phase III clinical trial for treating glioblastoma. Although cilengitide shows preclinical promise, the phase III trial demonstrates that cilengitide does not improve the effects of temozolomide on glioblastoma [161]. Randomized phase II study indicated that the effect of cilengitide and docetaxel on advanced non-small-cell lung cancer was similar [162], while it remains to know whether the addition of cilengitide to docetaxel may have clinical benefit. The α5β1-targeted peptide ATN-161 also showed no therapeutic benefits in clinical trials. Although other integrin-targeted agents including the anti-αVβ3 antibody etaracizumab (MEDI-522), the anti-α5β1 integrin antibody volociximab, the anti-αV antibodies intetumumab and abituzumab have been developed and assessed in phase I/II clinical trials, most of these trials are disappointing, and none of them have been tested in phase III trials. These data indicate that the roles of integrin in cancer may be much complex than expected. Since many integrins are also expressed in immune cells, we need to consider the effects of integrin inhibitors on immune surveillance. One study indicated that treatment with cilengitide might enhance the tumor-promoting M2 macrophages and reduce CD8(+) T cells [163]. It remains to know whether combination of integrin and immune checkpoints inhibitors has clinical benefits. In addition, low dose of cilengitide may paradoxically induce angiogenesis [164]. The pure αVβ3 antagonists TDI-4161 and TDI-3761 do not have such paradoxical effects [164]. It warrants further studies to determine the anticancer effects of these agents.

ILK is a critical regulator intracellular integrin signaling. Several ILK inhibitors have been developed and evaluated in preclinical studies. Recently, the tripeptides that mimic a fragment of alpha parvin, one of the ILK-interacting proteins, have been generated to interfere with ILK activity [165]. Other ILK inhibitors include N-methyl-3-(1-(4-(piperazin-1-yl)phenyl)-5-(4′-(trifluoromethyl)-[1,1′-biphenyl]-4-yl)-1H-pyrazol-3-yl)propanamide and QLT0267 [166, 167]. Preclinical studies have demonstrated that N-methyl-3-(1-(4-(piperazin-1-yl)phenyl)-5-(4′-(trifluoromethyl)-[1,1′-biphenyl]-4-yl)-1H-pyrazol-3-yl)propanamide and QLT-0267 have anticancer activities in vitro and in vivo. So far, no ILK inhibitors have been tested in clinical trials for cancer therapy.

### Preclinical and clinical development of YAP/TAZ-targeted agents

YAP and TAZ are important mechanotransducers that mediate the pro-tumor effects of ECM stiffening, although ECM rigidity may also have YAP/TAZ-independent effects. Verteporfin has been widely used as a YAP inhibitor and photosensitizer. Preclinical studies have demonstrated that verteporfin can effectively inhibit a various types of cancer [168]. Phase I/II clinical studies of photodynamic therapy with verteporfin or liposomal verteporfin (Visudyne) has been completed or being conducted in patients with recurrent high-grade EGFR-mutated glioblastoma or advanced pancreatic carcinoma [169] (Table 1).

YAP/TAZ often interacts with the TEA domain (TEAD) family proteins to regulate target genes expression. Pharmacological inhibition of YAP/TAZ-TEAD interaction is an approach to suppress YAP/TAZ signaling. K-975 is a selective TEAD inhibitor that binds to a cysteine residue in the palmitate-binding pocket of TEAD and inhibits YAP/TAZ-TEAD interactions [170]. Preclinical study demonstrates that K-975 can inhibit malignant pleural mesothelioma [170]. Flufenamic acid is another disruptor of YAP-TAZ/TEAD interaction. In addition, many compounds that target different pockets in TEAD to block YAP-TAZ-TEAD interactions have been developed. Of note, YAP and TAZ are not only transcriptional coactivators of TEAD, but also the coactivators of AP1 and STAT3 [171]. Hence, inhibition of YAP/TAZ-TEAD interactions may be not enough to abrogate YAP-TAZ signaling. An alternative approach is reducing YAP/TAZ protein levels by targeted protein degradation using proteolysis targeting chimeras (PROTACs) [172]. The PROTAC technology has already shown promise in cancer therapy. Some of the small molecule PROTACs have been evaluated in phase I clinical trials.

## Conclusions and perspectives

ECM stiffening coupled with ECM remodeling constitutes a vicious cycle that drives cancer progression. Increased ECM stiffness triggers mechanotransducing signal to stimulate the secretion of MMP from cancer and stromal cells. Elevated MMP activity promotes the degradation and reorganization of ECM components. Hence, ECM stiffness may be highly dynamic in cancer. The mechanotransduction-linked ECM remodeling is critical for the activation of cancer-associated stromal cells, tumor angiogenesis, immune evasion, tumor cells migration and invasion. ECM stiffness-related mechanical cues impinge on the cytoskeletal contractility of tumor and stromal cells. While integrins and focal adhesion dynamics are key mediators of ECM stiffening-induced cancer progression, it remains to identify other players in responding to increased ECM stiffness and enabling the mechanosignal transduce to all components in the tumor. Meanwhile, it warrants further studies to determine the mechanisms underpinning the regulation of cancer microenvironment, immune surveillance and cancer metastasis by ECM stiffness.

Given the important roles of ECM stiffness in cancer progression, detection of tumor stiffness may help to predict the prognosis of cancer patients. Noninvasive measurement of tissue stiffness can be achieved by shear wave elastography, magnetic resonance elastography and transient elastography. These techniques have demonstrated the correlation between tissue or tumor stiffness and clinicopathological characters. In addition, previous study indicates that collagen density is positively correlated with ECM stiffness [173]. While total collagen and immature collagen cross-links can be measured by mass spectroscopy-based techniques, the density and organization of collagen in tissues can be detected by picrosirius red staining viewed under polarized light microscopy [174]. It warrants further studies to determine the utility of elastography and measurements of collagen density in cancer diagnosis, staging, classification and prognostication.

The biophysical effects of ECM stiffness on cancer may interfere with drug delivery and the sensitivity to anticancer agents. Hence, detection of tumor stiffness may help stratification of patients for therapy. Collagenase can be utilized to directly deplete collagen, reduce ECM stiffness, improve drug penetration and sensitivity in tumor. However, there is concern about the safety of systemic collagenase treatment. The safety and bioavailability of recombinant collagenase may be improved by taking advantage of advanced biomaterials and drug delivery technologies [175]. An alternative approach is suppressing collagen synthesis and assembly. Inhibitors of TGFβ, integrins and YAP/TAZ can reduce collagen synthesis. Except for inhibition of collagen synthesis and deposition, Piezo, integrins and YAP/TAZ inhibitors can also block ECM stiffness-induced mechanotransduction. Some of these potential therapeutic avenues have been translated into clinical trials. While the results of integrins inhibitors in clinical trials are largely disappointing, we still expect that encouraging results may emerge from other pipelines such as the Hippo/YAP pathway inhibitors. Of note, there may be many obstacles and challenges for targeting ECM stiffness in cancer, due to the complex roles of ECM in cancer progression and the dynamic nature of ECM remodeling. Previous study indicated that transient mechano-intervention by short-term ROCK inhibition might improve the effect of chemotherapy on pancreatic carcinoma [176]. It warrants further studies to determine whether transient or prolonged ablation of ECM stiffening or remodeling is optimal for improving therapeutic efficacy in different types of cancer. The development of spatial-temporally controllable procedures to reverse ECM stiffening holds promise in improving chemotherapy efficacy [177]. As our knowledge of tumor matrix biology expands, we look forward to more targets being identified and more promising drugs being developed for cancer therapy.

## Data Availability

Not applicable.

## Abbreviations

CAF: Cancer-associated fibroblast
ECM: Extracellular matrix
EGFR: Epidermal growth factor receptor
EMT: Epithelial-mesenchymal transition
FAK: Focal adhesion kinase
ILK: Integrin-linked kinase
LH2: Lysyl hydroxylase 2
LOX: Lysyl oxidases
MMP: Matrix metalloproteinase
MSC: Mesenchymal stem cells
PROTACs: Proteolysis targeting chimeras
ROCK: Rho-associated protein kinase
SPARC: Secreted proteome acidic and rich in cysteine
TGFβ: Transforming growth factor β
TRPV4: Transient receptor potential vanilloid 4
